# Calcium Homeostasis and Psychiatric Disorders: A Mendelian Randomization Study

**DOI:** 10.3390/nu15184051

**Published:** 2023-09-19

**Authors:** Miaomiao Jiang, Weiheng Yan, Xianjing Li, Liyang Zhao, Tianlan Lu, Dai Zhang, Jun Li, Lifang Wang

**Affiliations:** 1 National Clinical Research Center for Mental Disorders (Peking University Sixth Hospital), NHC Key Laboratory of Mental Health, Peking University Sixth Hospital, Peking University Institute of Mental Health, Peking University, Beijing 100191, China; miao_mj@hsc.pku.edu.cn (M.J.); 2111110571@stu.pku.edu.cn (X.L.); 1911210694@pku.edu.cn (L.Z.); tllu@bjmu.edu.cn (T.L.); daizhang@bjmu.edu.cn (D.Z.); 2 Children’s Hospital Capital Institute of Pediatrics, Chinese Academy of Medical Sciences & Peking Union Medical College, Beijing 100020, China; yanwh@student.pumc.edu.cn; 3 Guangdong Key Laboratory of Mental Health and Cognitive Science, Institute for Brain Research and Rehabilitation (IBRR), South China Normal University, Guangzhou 510631, China

**Keywords:** serum 25-hydroxyvitamin D levels, psychiatric disorders, schizophrenia, Mendelian randomization study

## Abstract

Observational studies have investigated the impact of calcium homeostasis on psychiatric disorders; however, the causality of associations is yet to be established. Bidirectional Mendelian randomization (MR) analysis of calcium homeostasis hormones was conducted on nine psychiatric disorders. Calcium, serum 25-hydroxyvitamin D levels (25OHD), parathyroid hormone, and fibroblast growth factor 23 are the major calcium homeostasis hormones. The causality was evaluated by the inverse variance weighted method (IVW) and the MR Steiger test, while Cochran’s Q test, the MR-Egger intercept test, funnel plot, and the leave-one-out method were used for sensitivity analyses. Bonferroni correction was used to determine the causative association features (*p* < 6.94 × 10^−4^). Schizophrenia (SCZ) was significantly associated with decreased 25OHD concentrations with an estimated effect of −0.0164 (*P*_random-effect IVW_ = 2.39 × 10^−7^). In the Multivariable MR (MVMR) analysis adjusting for potentially confounding traits including body mass index, obesity, mineral supplements (calcium, fish oil, and vitamin D) and outdoor time (winter and summer), the relationship between SCZ and 25OHD remained. The genetically predicted autism spectrum disorder and bipolar disorder were also nominally associated with decreased 25OHD. This study provided evidence for a causal effect of psychiatric disorders on calcium homeostasis. The clinical monitoring of 25OHD levels in patients with psychiatric disorders is beneficial.

## 1. Introduction

Calcium and vitamin D play important roles in human health [1,2]. The effects of parathyroid hormone (PTH) include raising blood calcium levels, encouraging calcium absorption in the intestine with vitamin D, and reducing calcium excretion in the kidneys [3]. 25-hydroxyvitamin D (25OHD) is an indicator of vitamin D status, which is further converted in the kidneys to the active-form 1,25-dihydroxyvitamin D [4]. In the kidney, PTH stimulates, whereas fibroblast growth factor 23 (FGF23) represses 25OHD-1α-hydroxylation. FGF23 represses the activity of 25OHD-1α-hydroxylase, resulting in a decrease in the synthesis of 1,25-dihydroxyvitamin D [5]. FGF23 regulates the urinary calcium excretion and absorption of phosphate ions by the renal tubules [3,5]. In the distal convoluted tubule, FGF23 facilitates calcium reabsorption through the FGFR-Klotho complex [5]. These regulatory hormones interact closely to regulate calcium absorption, transport, and excretion.

Psychiatric disorder is one of the major public health challenges worldwide, ranking as the second most significant cause of premature death and disability [6]. Psychiatric disorders, due to their high prevalence, are the major causes of significant economic burden [7]. The pathophysiological mechanisms of psychiatric disorders are multifaceted [8]. Vitamin D and its metabolizing enzymes are expressed in various cerebral structures, and their deficiency has been linked to the development of psychiatric diseases [9]. In the observational analysis, higher concentrations of 25OHD were associated with a reduced risk of depression [10]. According to a prospective cohort study of the general population, a reduction in plasma 25OHD levels was linked to an increased risk for Alzheimer’s disease (AD) and vascular dementia [11]. Previous studies have found that low prenatal vitamin D levels may increase the risk of attention-deficit hyperactivity disorder (ADHD) or autism spectrum disorder (ASD) in offspring [12,13]. A meta-analysis of 19 studies has shown that individuals diagnosed with schizophrenia (SCZ) exhibited lower levels of serum vitamin D compared to the control group [14]. Children with ASD exhibit significantly lower vitamin D levels than their non-ASD siblings according to a study analyzing 25OHD in born sibling pairs [15]. Vitamin D supplementation reduces depressive symptoms and decreases the risk of developing psychotic-like symptoms, suggesting a potential therapeutic benefit for patients with psychiatric disorders [16,17,18]. However, the findings are not entirely consistent. Previously, a meta-analysis failed to find evidence supporting the improvement of depressive symptoms in adults through vitamin D supplementation [19]. Many observational studies have shown that low vitamin D status is common in individuals with ASD, SCZ or ADHD, and vitamin D supplementation improves the symptoms [20,21,22]. A previous meta-analysis demonstrated the beneficial impact of vitamin D supplements on ADHD [23]. In contrast, another study reported no significant effects of vitamin D supplementation on core symptoms of ASD [24]. A recent study revealed that after adjusting for age and years of education, there was an inverse association between FGF23 levels in cerebrospinal fluid and impulsivity scores [25]. In addition, some studies have found increased levels of FGF23 in individuals with postpartum depression, alcoholics, and patients with episodic sleep disorders [26,27,28]. However, whether the observed association is causal is still unknown.

Mendelian randomization (MR) uses genetic variants as instrumental variables to establish causality in exposure–outcome associations while avoiding reverse causality [29]. MR is more appropriate to detect the long-term causal effects of exposure on outcomes due to its utilization of naturally occurring genetic variation as instrumental variables, which are randomly allocated during conception and have lifelong effects [29]. Multivariable MR (MVMR) is an extension of MR that is used to assess whether the associations remain after controlling for potential confounders [30]. In comparison to observational studies, MVMR enables the determination of causal relationships by minimizing biases caused by confounding factors. We performed a bidirectional two-sample MR analysis and MVMR study to investigate the causal associations between calcium homeostasis and psychiatric disorders.

## 2. Materials and Methods

### 2.1. Study Design

**Figure 1:**
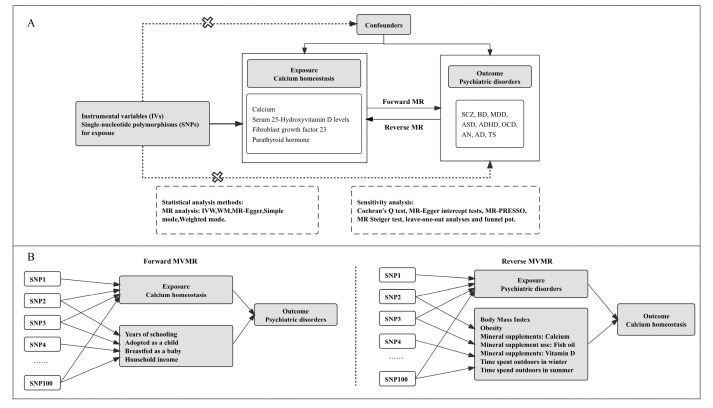
Study flow diagram. (**A**) Univariable Mendelian Randomization. The dashed lines with the symbol “X” represent the putative pleiotropic or direct causal effects between variables that might violate MR assumptions. (**B**) Multivariable MR (MVMR) allows an additional variable, besides the main exposure. E.g., whether there is a causal effect of schizophrenia on 25OHD after considering body mass index, mineral supplements, or outdoor activities. ASD, autism spectrum disorder; ADHD, attention-deficit/hyperactivity disorder; SCZ, schizophrenia; BD, bipolar disorder; MDD, major depressive disorder; AD, Alzheimer’s disease; AN, anorexia nervosa; OCD, obsessive–compulsive disorder; TS, Tourette syndrome; IVW, inverse-variance weighted; WM, weighted median; MR, Mendelian randomization; MR-PRESSO, MR pleiotropy residual sum and outlier.

A bidirectional MR and an MVMR design were used to detect the causal effects between genetically predicted calcium, 25OHD, PTH, FGF23, and nine psychiatric disorders (Figure 1). The psychiatric disorders included AD, ADHD, anorexia nervosa (AN), ASD, bipolar disorder (BD), major depressive disorder (MDD), obsessive–compulsive disorder (OCD), Tourette syndrome (TS), and SCZ. The validity of genetic instruments is based on three critical principles: (1) the single nucleotide polymorphisms (SNPs) from genome-wide association studies (GWAS) applied as instrumental variables (IVs) were related to exposures; (2) IVs must not be associated with confounders; (3) IVs should not affect the outcome directly but only through their respective exposure traits [31] (Figure 1A).

### 2.2. Data Extraction

The summary statistics were collected from Psychiatric Genomics Consortium (PGC: https://www.med.unc.edu/pgc/, (accessed on 3 April 2023)) and GWAS summary data (https://gwas.mrcieu.ac.uk/, (accessed on 11 April 2023)). Detailed information on the GWAS datasets is provided in Table 1. Only summarized data from the European population were utilized to minimize population heterogeneity bias. 

The summary statistics for calcium homeostasis, including serum calcium, 25OHD, FGF23, and PTH, were extracted from five different GWASs. According to Neale Lab genome-wide association meta-analysis (http://www.nealelab.is/uk-biobank/, (accessed on 11 April 2023)), the GWAS summary statistics for calcium was based on 315,153 samples from UK Biobank [32]. This GWAS adjusted for age, sex, and the top twenty principal components. The genetic predictors for serum 25OHD were obtained from the largest GWAS (n = 443,734) [33], while the summary statistics for the other factors were derived from studies with n = 21,758 for FGF23 [34], and n = 3301 for PTH [35]. The sample sizes of nine psychiatric disorders were as follows: AD [36] (71,880 cases and 383,378 controls), ADHD [37] (38,691 cases and 38,691 controls), AN [38] (3495 cases and 10,982 controls), ASD [39] (18,381 cases and 27,969 controls), BD [40] (20,352 cases and 31,358 controls), MDD [41] (170,756 cases and 329,443 controls), OCD [42] (2688 cases and 7037 controls), SCZ [43] (52,017 cases and 75,889 controls), and TS [44] (4819 cases and 9488 controls) (Table 1). Informed consent and ethics approval were obtained for each of the original studies.

**Table 1:** Detailed information regarding studies and datasets used in the present study.

Exposure or Outcome	Reference	Participants	Web Source
Serum 25-Hydroxyvitamin D levels	PMID: 32059762 [33]	443,734 individuals	* ebi-a-GCST010144
Calcium	PMID: 34662886 [32] ^a^	315,153 individuals	* ukb-d-30680_irnt
Fibroblast growth factor 23	PMID: 33067605 [34]	21,758 individuals	* ebi-a-GCST90012022
Parathyroid hormone	PMID: 29875488 [35]	3301 individuals	* prot-a-2431
Alzheimer’s disease	PMID: 30617256 [36]	71,880 cases and 383,378 controls	PGC
Attention-deficit/hyperactivity disorder	PMID: 29325848 [37]	38,691 cases and 38,691 controls	PGC
Anorexia nervosa	PMID: 28494655 [38]	3495 cases and 10,982 controls	PGC
Autism spectrum disorder	PMID: 30804558 [39]	18,381 cases and 27,969 controls	PGC
Bipolar disorder	PMID: 31043756 [40]	20,352 cases and 31,358 controls	PGC
Major depressive disorder	PMID: 30718901 [41]	170,756 cases and 329,443 controls	PGC
Obsessive–compulsive disorder	PMID: 28761083 [42]	2688 cases and 7037 controls	PGC
Schizophrenia	PMID: 35396580 [43]	52,017 cases and 75,889 controls	PGC
Tourette syndrome	PMID: 30818990 [44]	4819 cases and 9488 controls	PGC

^a^ http://www.nealelab.is/uk-biobank/, (accessed on 11 April 2023); Output from GWAS pipeline using Phesant-derived variables from UK Biobank. * https://gwas.mrcieu.ac.uk/datasets/, (accessed on 11 April 2023); PGC: https://pgc.unc.edu/, (accessed on 3 April 2023).

### 2.3. Selection of the Instrumental Variables (IVs)

In MR analyses, we used a *p* < 1 × 10^−5^ threshold to select SNPs as instrumental variables. This approach was adopted to increase the number of SNPs available for sensitivity analyses. SNPs with high linkage disequilibrium were excluded with a strict r^2^ cutoff of 0.0001 and a clumping window greater than 10,000 kb [45]. SNPs with indirect effects were removed if they were associated (*p*-value < 0.001) with the outcomes. The selection of IVs must fulfill three critical assumptions: the SNPs are highly associated with the exposure but not with the outcome or confounding factors. The F-statistic values below a threshold of 10 indicate a higher degree of bias [46]. We calculated the F statistic values using the formula *F* = ((*R*^2^/(1 − *R*^2^)) × ((N − K − 1)/K)] to assess instrument strength for the forward and reverse MR pairs. Briefly, the *R*^2^ represents the explained variance of genetic instruments, K represents the total number of IVs included in each MR analysis, and N represents the sample size of the exposure GWAS data [47].

### 2.4. Two-Sample Univariable MR Analysis

The main analysis was conducted using the random effects IVW method, which provides precise causal estimates while adjusting for heterogeneity of IVs [48]. Weighted median (WM) [48] and MR-Egger [49] were also employed as supplementary approaches to investigate the causal correlation. MR-Egger is an adaption of Egger regression, and the slope coefficient of the Egger regression can be used for causal effect estimation [48]. The weighted median (WM) method is used to combine data from multiple genetic variants into a single causal estimate. This estimator is consistent when as much as 50% of the data is derived from invalid IVs [49]. The significant (*p* < 0.05) results of MR-IVW were considered positive indicating a meaningful association. Furthermore, the direction of the MR analysis results (beta value) remained consistent across all three methods (IVW, MR-Egger and WM). [50]. Bonferroni’s correction for multiple testing was conducted to estimate *p*-values. A *p*-value < 6.94 × 10^−4^ (0.05/36/2; 2 denotes both forward and reverse MR tests) was a strong evidence of a causal association. The beta and 95% confidence intervals (CIs) were used to present the causal correlation between psychiatric disorders and calcium homeostasis. For the other direction, the casual estimate was presented as an odds ratio (OR) and 95% CIs.

### 2.5. Sensitivity Analysis

Sensitivity analysis was conducted to identify any horizontal pleiotropy that would contradict the main MR hypothesis. The MR analyses utilized the TwoSampleMR package (version 0.5.6) [51]. A leave-one-out analysis was conducted to detect the causal estimates that may be affected by a single SNP. The global MR pleiotropy residual sum and outlier (MR-PRESSO) (https://github.com/rondolab/MR-PRESSO/, (accessed on 11 April 2023)) test was introduced to explore the possible outlier SNPs [52]. Next, we investigated the possibility of directional pleiotropy and heterogeneity using MR-Egger regression and the Cochran Q test [53]. Additionally, we conducted the MR-Steiger directionality test to assess the potential causal correlation between the assumed exposure and potential outcomes.

### 2.6. MVMR Analysis

The multivariable Mendelian randomization (MVMR) was used to verify direct causality between psychiatric disorders and calcium homeostasis. MVMR-IVW, MVMR-Egger, MVMR-Robust, MVMR-median, and the least absolute shrinkage and selection operator (LASSO) were used to determine direct causality [54]. If at least one of these five methods yields a significant result, it is considered that the causal relationship still exists even after multivariable adjustment. To consider the potential for genetic confounding, traits such as body mass index (BMI), obesity, mineral supplements (calcium), mineral supplement use (fish oil), mineral supplements (vitamin D), time spent outdoors in winter, time spend outdoors in summer, years of schooling, adopted as a child, breastfed as a baby, and household income were examined. For each multivariable analysis, we added each genetic confounding separately. The sample sizes for these factors were adopted from their respective GWASs and ranged from 64,949 to 766,345 individuals (Supplementary Table S1).

## 3. Results

### 3.1. Genetic Instruments Selected in MR

The flowchart of the study design is illustrated in Figure 1. The details of IVs used in the MR analysis are listed in Supplementary Table S2. All F statistic values were > 10, as reported in Supplementary Table S3. 

### 3.2. Causal Effect of Genetically Predicted Calcium Homeostasis on Psychiatric Disorders

**Figure 2:**
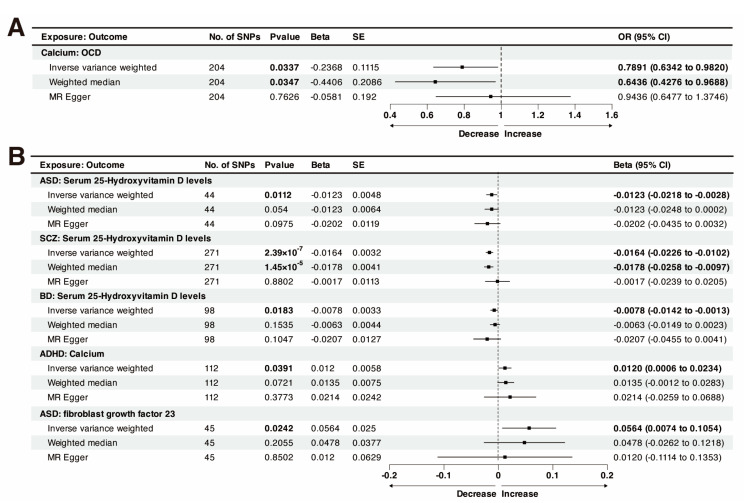
Forest plot shows the causalities of the psychiatric disorders and calcium homeostasis. (**A**) Associations between genetically predicted calcium homeostasis on psychiatric disorders. (**B**) Associations between genetically predicted psychiatric disorders on calcium homeostasis. No, number; SNP, single nucleotide polymorphism; beta, genetic effect size from the exposure GWAS data; SE, standard error of effect size.

According to the IVW results, genetically predicted Calcium was nominally associated with lower-odds OCD (odds ratio (OR) = 0.7891, 95% CI: 0.6342–0.9820; *p* = 0.0337; *p*-Egger intercept = 0.2540; Figure 2A). The IVW analysis showed that the genetically predicted serum 25OHD levels were associated with lower-odds ASD (OR = 0.7520, 95% CI: 0.5889–0.9602; *p* = 0.0223). However, different MR analysis methods were contradictory, and the MR-Egger test indicated that 25OHD is associated with higher-odds ASD (OR = 1.8189, 95% CI: 0.6386–5.1812; *p* = 0.2644), although the association was not statistically significant. The direction of the causal relationship between FGF23 and ASD appears contradictory across different methods. While the MR-Egger method suggests an increased risk of ASD associated with FGF23, both WM and IVW methods indicate a protective effect. These results did not meet the criteria for positive results in this study. The detailed two-sample MR results can be viewed in Supplementary Table S4.

### 3.3. Causal Effect of Genetically Predicted Psychiatric Disorders on Calcium Homeostasis

**Figure 3:**
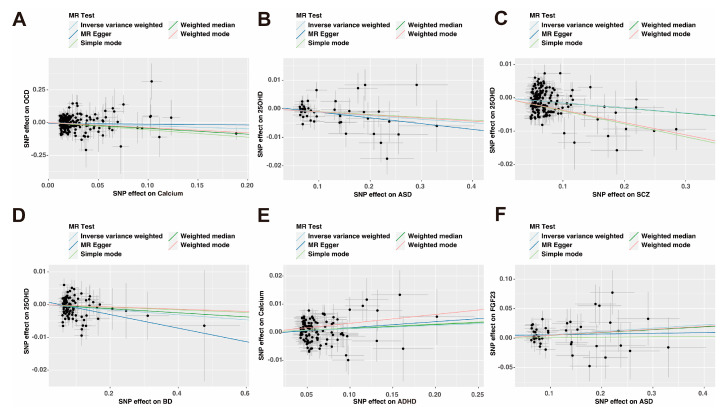
Scatterplot of the effect of the psychiatric disorders and calcium homeostasis. An association between the psychiatric disorders and calcium homeostasis through five Mendelian randomization (MR) methods (**A**–**F**). The slope value equals the b-value calculated using the five methods and represents the causal effect.

In the reverse-direction MR study, the putative causal effects of psychiatric disorders on calcium homeostasis were estimated (Figure 2B and Figure 3). As shown by the funnel plot, the effect size variation around the point estimate was symmetrical after excluding outliner SNPs (Supplementary Figures S1–S6). The results of leave-one-out analysis confirmed that single SNPs did not affect the causal association (Supplementary Figure S7).

The Bonferroni-corrected *P* threshold of 6.94 × 10^−4^ obtained from 72 tests identified a significant causal correlation between SCZ and low levels of 25OHD. To reduce heterogeneity in causality, the SNPs identified as outliers (rs10873538, rs2252074, rs6690619, rs6798742, and rs9304548) were removed from the analysis based on the results of the MR-PRESSO test (Supplementary Figure S4). The causal effect estimate was −0.0164 (95% CI: −0.0226 to −0.0102, *p*_random-effect IVW_ = 2.39 × 10^−7^), which was consistent in the weighted-median method (beta = −0.0178, 95% CI: −0.0258 to −0.0097, *p* = 1.45 × 10^−5^; *p*-Egger intercept = 0.1764). The MR-Egger intercept test did not provide any evidence of directional pleiotropy between SCZ and 25OHD. The current study detected heterogeneity based on Cochran’s Q test, indicating that a random effects IVW model was appropriate. Furthermore, after excluding outlier SNPs, the funnel plot showed symmetric variation in effect size around the point estimate (Supplementary Figure S4B). The results of the “leave-one-out” method confirmed that single SNPs did not affect the causal association (Supplementary Figure S7D). The MR Steiger directionality test results indicated that our estimation of causal direction is accurate. 

Notably, ASD and BD showed a potential decrease in 25OHD levels (Figure 3B,D). *p*-value < 0.05 indicated statistical significance for genetic correlations. The results of IVW analysis showed a nominally causal effect of ASD on decreased 25OHD (beta = −0.0123, 95% CI: −0.0218 to −0.0028, *p* = 0.0112; *p*-Egger intercept = 0.4721). The directions of the estimates from the WM and MR-Egger tests were the same as those from the IVW method. Following the exclusion of one outlier SNP (rs4301023), the current study found an association between BD and 25OHD levels (beta = −0.0078, 95% CI: −0.0142 to −0.0013, *p* = 0.0183; *p*-Egger intercept = 0.2916). MR-Steiger directional test supported the hypothesis (*p*-Steiger test= 5.96 × 10^−10^), and multiple sensitivity analyses suggested robust causal correlations (Table 2). Furthermore, genetically predicted ASD has a causal effect on FGF23 (beta = 0.0564, 95% CI: 0.0074–0.1054, *p* = 0.0242; *p*-Egger intercept = 0.4457), and the causal direction is accurate (*p*-Steiger test = 8.73 × 10^−39^). The Cochran-Q-test-derived *p*-value was 0.5939, indicating no obvious heterogeneity (Table 2).

After excluding outlier SNPs (rs34008721, and rs139950543), ADHD was nominally associated with an increase in calcium levels (beta = 0.0120, 95% CI: 0.0006–0.0234, *p*_random-effect IVW_ = 0.0391; *p*-Egger intercept = 0.6884). Cochran’s Q test indicated heterogeneity in the results, thus prioritizing “random–IVW” methods. MR-Steiger directional test supported our hypothesis (*p*-Steiger test < 0.001). 

### 3.4. Multivariable Mendelian Randomization

**Figure 4:**
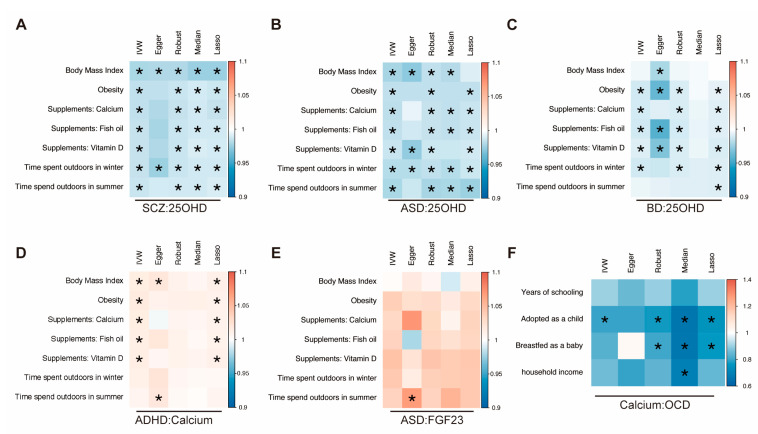
Multivariable MR (MVMR) associations of psychiatric disorders with calcium homeostasis. (**A**) The causal effect of SCZ on 25OHD through MVMR analysis. (**B**) The causal effect of ASD on 25OHD through MVMR analysis. (**C**) The causal effect of BD on 25OHD through MVMR analysis. (**D**) The causal effect of ADHD on calcium through MVMR analysis. (**E**) The causal effect of ASD on FGF23 through MVMR analysis. (**F**) The causal effect of calcium on OCD through MVMR analysis.For each multivariable analysis, we added each genetic confounding separately. If at least one of these five methods yields a significant result, it is considered that the causal relationship still exists even after multivariable adjustment. SCZ, schizophrenia; ASD, autism spectrum disorder; ADHD, attention-deficit/hyperactivity disorder; BD, bipolar disorder; OCD, obsessive–compulsive disorder; 25OHD, serum 25-Hydroxyvitamin D levels; FGF23, fibroblast growth factor 2; IVW, inverse-variance weighted; egger, MR-Egger; median, weighted median; lasso, least absolute selection, and shrinkage operator; MR, Mendelian randomization. * *p* < 0.05.

The detailed MVMR results are presented in Supplementary Tables S5–S10. In the multivariable MR models, the results of the associations between SCZ and 25OHD were also robust when adjusted for BMI (adjusted beta = −0.0206, 95% CI: −0.0299 to −0.0112, *p*_IVW_ = 1.56 × 10^−5^; adjusted beta = −0.0197, 95% CI: −0.0293 to −0.0100, *p*_Robust_ = 6.17 × 10^−5^; adjusted beta = −0.0246, 95% CI: −0.0316 to −0.0177, *p*_LASSO_ = 3.51 × 10^−12^) and obesity (adjusted beta = −0.0151, 95% CI: −0.0214 to −0.0088, *p*_IVW_ = 2.72 × 10^−6^; adjusted beta = −0.0139, 95% CI: −0.0205 to −0.0073, *p*_Robust_ = 3.91 × 10^−5^; adjusted beta = −0.0130, 95% CI: −0.0177 to −0.0083, *p*_LASSO_ = 7.23 × 10^−8^). Even after adjusting for mineral supplements (calcium, fish oil, and vitamin D) and outdoor time (winter and summer), the significant association between SCZ and 25OHD persisted (*p*_IVW_ < 6.94 × 10^−4)^, indicating that SCZ was an independent risk factor for decreased 25OHD (Figure 4A and Supplementary Table S7). MVMR analysis also indicated that ASD was a potential independent risk factor for decreased 25OHD (*p*_IVW_ < 0.05) (Figure 4B). 

In analyses using MVMR-Egger, BD was associated with a decreased 25OHD level when adjusted for BMI (adjusted beta = −0.0039, 95% CI: −0.0150 to −0.0072, *p*_Egger_ = 0.0143) and time spent outdoors in summer (adjusted beta = −0.0082, 95% CI: −0.0141 to −0.0022, *p*_LASSO_ = 0.0070). Even after adjusting for mineral supplements (calcium, fish oil, and vitamin D), the relationship between BD and 25OHD persisted when using the IVW method (*p*_IVW_ < 0.05) (Figure 4C). 

The previously established causal relationship between ADHD and calcium result became insignificant after adjusting for outdoor time. No genetic association of ASD with FGF23 was found after correction for obesity and BMI. In addition, calcium was not associated with a reduced risk of OCD in MVMR analysis after correcting for years of schooling, breastfed as a baby, and household income (Figure 4).

## 4. Discussion

The primary objective of our study was to use bidirectional two-sample MR and MVMR analysis to explore the causal associations between calcium homeostasis markers (calcium, 25OHD, PTH, and FGF23) and nine psychiatric disorders. The present study provides valuable insights into the correlation between psychiatric disorders and calcium homeostasis. MVMR analysis indicated that psychiatric disorders (SCZ, ASD, and BD) were potential risk factors for decreased 25OHD.

After Bonferroni’s correction, the reverse MR analysis indicated that genetically predicted SCZ was significantly associated with decreased 25OHD. Further MVMR analysis indicated that the relationship remains robust even after adjusting for another seven variables. We used the latest SCZ summary statistical data (n = 127,906) and the largest serum 25OHD GWAS dataset (n = 443,734). This result was consistent with other observational studies. A cross-sectional study showed that SCZ patients had higher levels of C-reactive protein (CRP) and lower levels of 25OHD than controls [55]. Low serum 25OHD levels have been linked to high CRP levels in Mendelian randomization studies, and raising vitamin D status may help to reduce inflammation [56]. A case–control study found that both low and high concentrations of neonatal vitamin D are associated with an increased risk of SCZ [57]. The study found that first-episode psychosis (FEP) patients had low levels of 25OHD, and low 25OHD level correlated with psychiatric symptoms [58]. The connection between early nutrition and psychiatric disorders garnered significant attention. A Finnish birth cohort study showed that men who received vitamin D supplements containing at least 2000 IU during the first year of life had a lower risk of developing SCZ [22]. In a recent study using a phenome-wide (PheWAS)-based Mendelian randomization, higher weighted genetic risk scores (GRS) for SCZ were associated with low 25OHD levels and calcium content [59]. In previous studies, vitamin D was negatively associated with MDD and SCZ PRS [60]. In individuals with schizophrenia, the reduction in physical activity due to medication side effects may contribute to an increased risk of vitamin D deficiency [59].

The current study showed a nominally association between genetically predicted ASD and decreased 25OHD levels. A cross-sectional study suggested the mean level of serum 25OHD levels was lower in the ASD group compared to the non-ASD group after adjusting for age and sex [61]. The primary conversion steps of vitamin D absorption and metabolism into 25OHD are predominantly catalyzed by the microsomal enzyme CYP2R1 which belongs to the cytochrome P-450 (CYP) family of enzymes and is mainly located in the liver [62]. Oral supplementation with calcifediol (25OHD3) instead of vitamin D alone could enhance the intake of the vitamin [63]. Neural development and normal brain homeostasis are significantly affected by early nutrition, with a crucial role of vitamin D in the development of the central nervous system, as supported by multiple studies [64,65]. A previous study found a correlation between low serum 25-OHD levels and increased severity of symptoms in patients, with vitamin D supplementation resulting in improved stereotypical behavior and attention span [20]. Another earlier study identified a link between 25OHD and ADHD symptoms, and the use of vitamin D supplementation as adjunctive therapy to methylphenidate showed improvement in patient symptoms [21]. According to many observational reviews, low vitamin D status may be common in ASD or ADHD [66,67]. The current study also revealed a nominally association between BD and decreased 25OHD levels. Previous studies have shown that patients experiencing acute manic episodes have lower vitamin D serum concentrations compared to the healthy control group [68]. Vitamin D supplementation was associated with a reduction in both depressive and manic symptoms in patients with psychiatric disorders [69].

To the best of our knowledge, this is the most comprehensive and extensive MR study investigating the genetic association between calcium homeostasis and psychiatric disorders. Nevertheless, the present study has some limitations. Firstly, the GWAS studies included in this research are based on European populations, reducing the possibility of stratification bias. Different racial groups exhibit variations in vitamin D metabolism; African Americans tend to have low levels of VDBP and 25OHD without evidence of vitamin D deficiency [70]. However, it must be recognized that there may be racial/ethnic differences [70,71]. Therefore, including populations with different characteristics (such as race and age) in MR studies may provide different results. Secondly, the SNPs used in the analysis did not reach conventional genome-wide association significance thresholds. However, the study had no weak IVs according to the F statistics. Thirdly, we used the latest available GWAS with a maximum sample size in the data analysis; the field of genetic research has a high rate of updates, and more GWAS will be available in the future. The GWAS data used for calcium included in this study were obtained from Neale Lab, and we acknowledge the limitation of relying on data from a single cohort. In addition, the relationship between the calcium level or the 25OHD level and the risk of diseases may be nonlinear [72]. It is possible that we may be missing the true link between calcium levels and disease. We used the most recent GWAS data for our analysis, and the current MR analysis does not support a direct causal relationship between calcium levels and psychiatric disorders. It is important to note that findings from our MR study should not be interpreted as final results. Finally, our findings supported the existence of abnormalities in calcium and its regulating hormone levels in different psychiatric disorders. While interpreting the results, the instrumental variables not originating from GWAS focusing on children must be considered. The threshold for vitamin D supplementation and the amount required among individuals of different ages and weights must also be considered. In addition, large-scale studies are required to investigate the potential long-term impact of vitamin D supplementation in patients with psychiatric disorders. The choice between cholecalciferol (vitamin D3) and calcifediol (25OHD3) also needs to be considered [63]. Resolving these issues is imperative to gain insight into the clinical advantages of vitamin D supplementation. In future, randomized controlled trials can provide a more dynamic perspective on the relationship between calcium homeostasis and various psychiatric disorders.

## 5. Conclusions

In summary, this bidirectional MR study shows a significant correlation between SCZ and 25OHD reduction predicted by genetics. It also provides evidence for previous studies, such as abnormalities in calcium and its regulating hormone levels in different psychiatric disorders. Thus, monitoring the 25OHD level in patients with psychiatric disorders may be a good clinical practice.

## Figures and Tables

**Table 2:** Sensitivity analysis of the causal association.

Exposure: Outcome	F-Statistic	MR-Egger_ Intercept	Egger Intercept _Pval ^a^	IVW_ Cochrane_Q	Cochrane _Q_Pval ^b^	Steiger Test	Steiger Test _Pval ^c^
Calcium: OCD	76.1028	−0.0058	0.2540	206.4289	0.4198	TRUE	1.83 × 10^−11^
SCZ: 25OHD	255.2465	−0.0009	0.1764	497.7460	1.02 × 10^−15^	TRUE	<0.001
ASD: 25OHD	124.8705	0.0008	0.4721	52.9345	0.1425	TRUE	1.09 × 10^−191^
BD: 25OHD	168.0641	0.0011	0.2916	128.5298	0.0177	TRUE	5.96 × 10^−10^
ADHD: Calcium	108.0976	−0.0005	0.6884	163.5865	0.0009	TRUE	<0.001
ASD: FGF23	125.5161	0.0046	0.4457	41.1640	0.5939	TRUE	8.73 × 10^−39^

^a^ The MR-Egger intercept quantifies the effect of directional pleiotropy (*p* < 0.05, which means possible pleiotropy). ^b^ The Cochrane-Q test quantifies the effect of heterogeneity (*p* < 0.05, which means possible heterogeneity, thus prioritizing “random–IVW” methods). ^c^ MR-Steiger directionality test to assess the potential causal relationship. F-stat, conditional F-statistic; Q, Cochran’s Q statistics; 25OHD: Serum 25-Hydroxyvitamin D levels; FGF23: Fibroblast growth factor 23.

## Data Availability

The original contributions presented in the study are included in the article/Supplementary Material, further inquiries can be directed to the corresponding authors. Computing code can be available through corresponding authors.

## Supplementary Materials

The following supporting information can be downloaded at: https://www.mdpi.com/article/10.3390/nu15184051/s1, Figure S1: Funnel plot for calcium on OCD. Figure S2: Funnel plot for ASD on 25OHD. Figure S3: Funnel plot for ASD on FGF23. Figure S4: Funnel plot for SCZ on 25OHD. Figure S5: Funnel plot for BD on 25OHD. Figure S6: Funnel plot for ADHD on calcium. Figure S7: Leave-one-out analysis of putative causal effects. Table S1. Detailed information regarding studies and datasets used in MVMR analysis. Table S2: Instrumental variables used in MR analysis. Table S3: Power estimation of this Mendelian randomization analysis and sensitivity analysis of the causal association. Table S4: Association of genetically predicted calcium homeostasis with psychiatric disorders in univariable mendelian randomization. Table S5: Multivariable mendelian randomization (MVMR) estimation between calcium and OCD. Table S6: MVMR estimation between ASD and 25OHD. Table S7: MVMR estimation between SCZ and 25OHD. Table S8: MVMR estimation between BD and 25OHD. Table S9: MVMR estimation between ADHD and calcium. Table S10: MVMR estimation between ASD and FGF23.
