# Supplementary material for: Calcium Homeostasis and Psychiatric Disorders: A Mendelian Randomization Study
Source: Nutrients. 2023 Sep 19;15(18):4051. doi: 10.3390/nu15184051 (PMC10535008; doi:10.3390/nu15184051)
Supplement: Supplementary file 1 [file nutrients-15-04051-s001.zip › Supplementary Figures.pdf]

Supplementary Figures

Supplementary Figure S1. Funnel plot for calcium on OCD.

Supplementary Figure S2. Funnel plot for ASD on 25OHD.

Supplementary Figure S3. Funnel plot for ASD on FGF23.

Supplementary Figure S4. Funnel plot for SCZ on 25OHD.

Supplementary Figure S5. Funnel plot for BD on 25OHD.

Supplementary Figure S6. Funnel plot for ADHD on calcium.

Supplementary Figure S7. Leave-one-out analysis of putative causal effects.

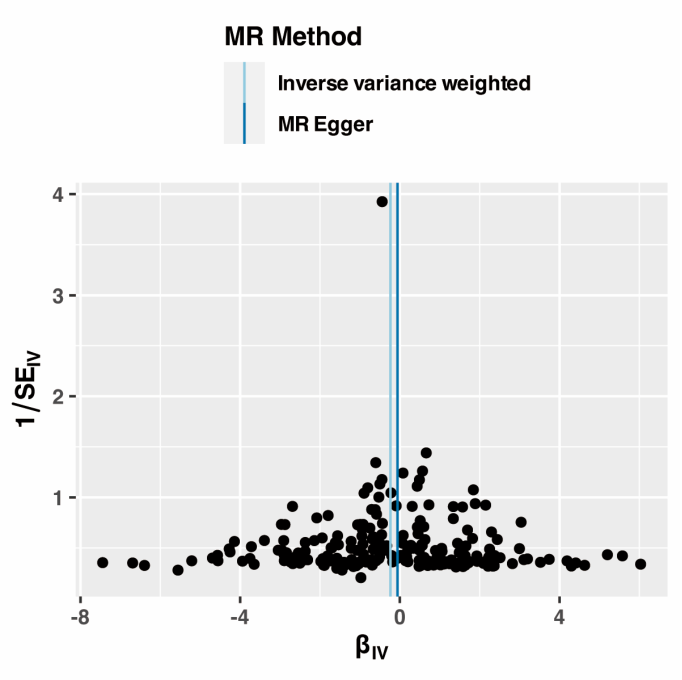

Supplementary Figure S1. Funnel plot for calcium on OCD.

Each point is a single nucleotide polymorphism. OCD, obsessive-compulsive disorder; SE, standard error.

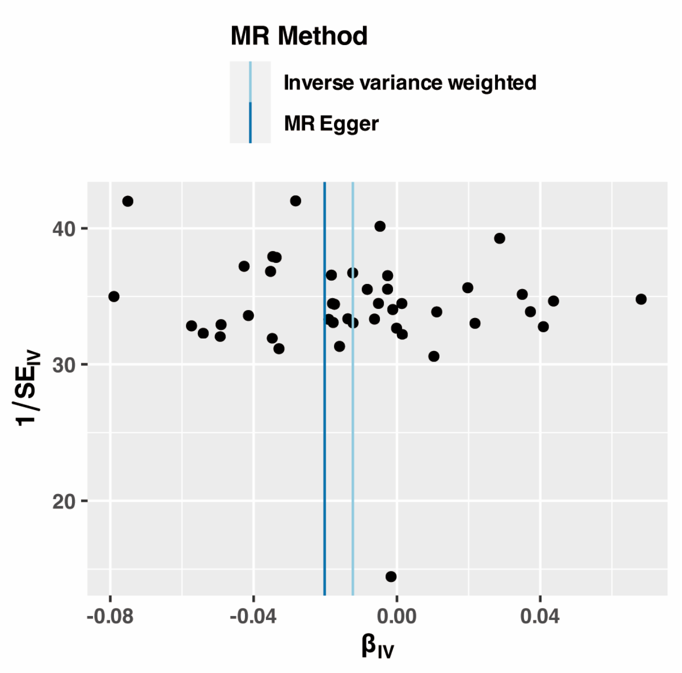

Supplementary Figure S2. Funnel plot for ASD on 25OHD.

Each point is a single nucleotide polymorphism. ASD, autism spectrum disorder; 25OHD, serum 25-Hydroxyvitamin D levels; SE, standard error.

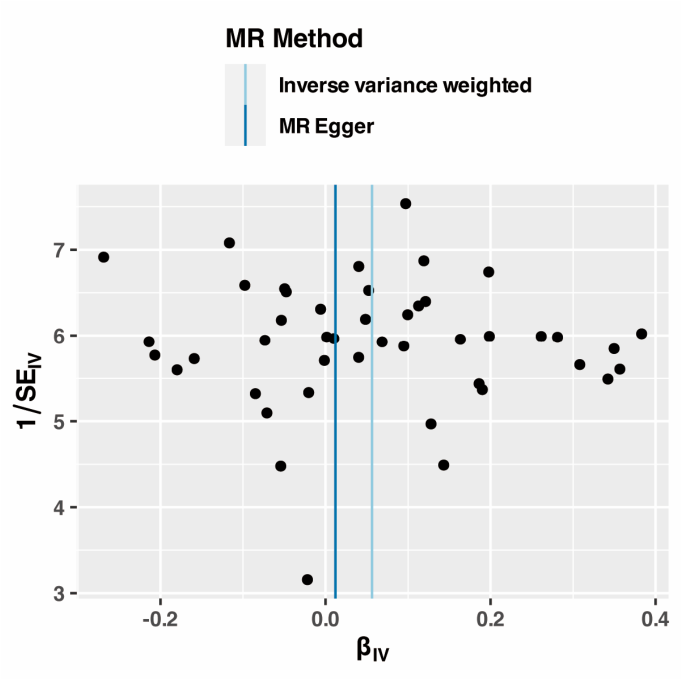

Supplementary Figure S3. Funnel plot for ASD on FGF23.

Each point is a single nucleotide polymorphism. ASD, autism spectrum disorder; FGF23, fibroblast growth factor 23; SE, standard error.

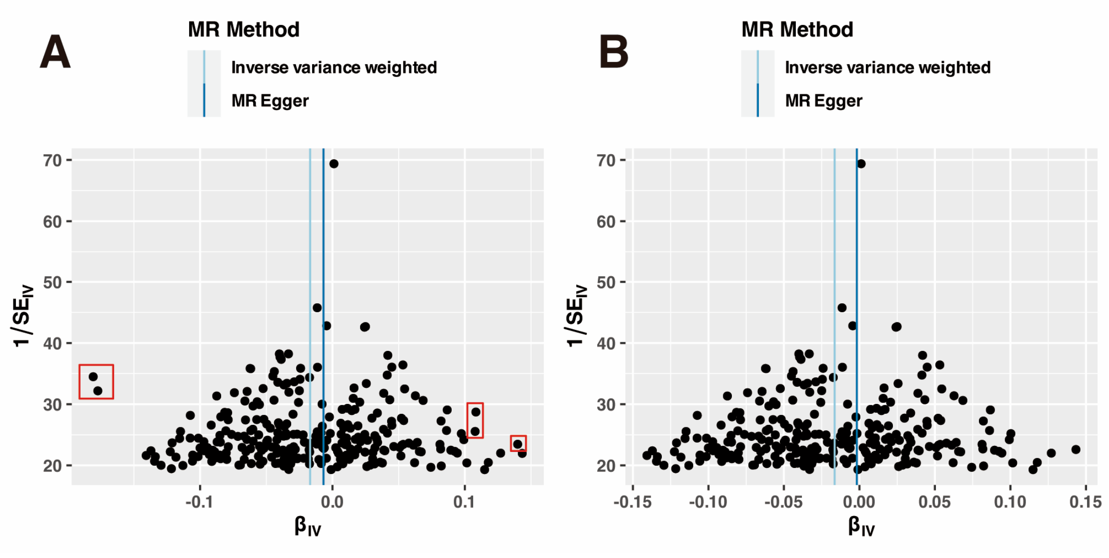

Supplementary Figure S4. Funnel plot for SCZ on 25OHD. (A) The funnel plot of SCZ-25OHD results before removal of SNPs. (B) The funnel plot of SCZ-25OHD results after removal of SNP rs10873538, rs2252074, rs6690619, rs6798742, and rs9304548.

The global MR pleiotropy residual sum and outlier (MR-PRESSO) test was introduced to explore the possible outlier SNPs. Each point is a single nucleotide polymorphism. SCZ, schizophrenia; 25OHD, serum 25-Hydroxyvitamin D levels; SE, standard error.

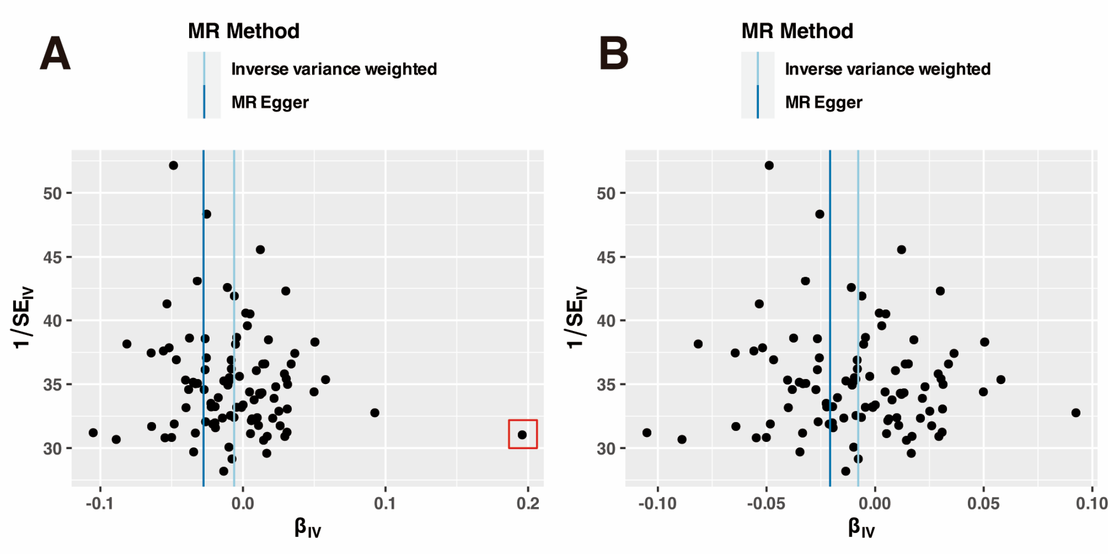

Supplementary Figure S5. Funnel plot for BD on 25OHD. (A) The funnel plot of BD-25OHD results before removal of SNPs. (B) The funnel plot of BD-25OHD results after removal of SNP rs4301023.

The global MR pleiotropy residual sum and outlier (MR-PRESSO) test was introduced to explore the possible outlier SNPs. Each point is a single nucleotide polymorphism. BD, bipolar disorder; 25OHD, serum 25-Hydroxyvitamin D levels; SE, standard error.

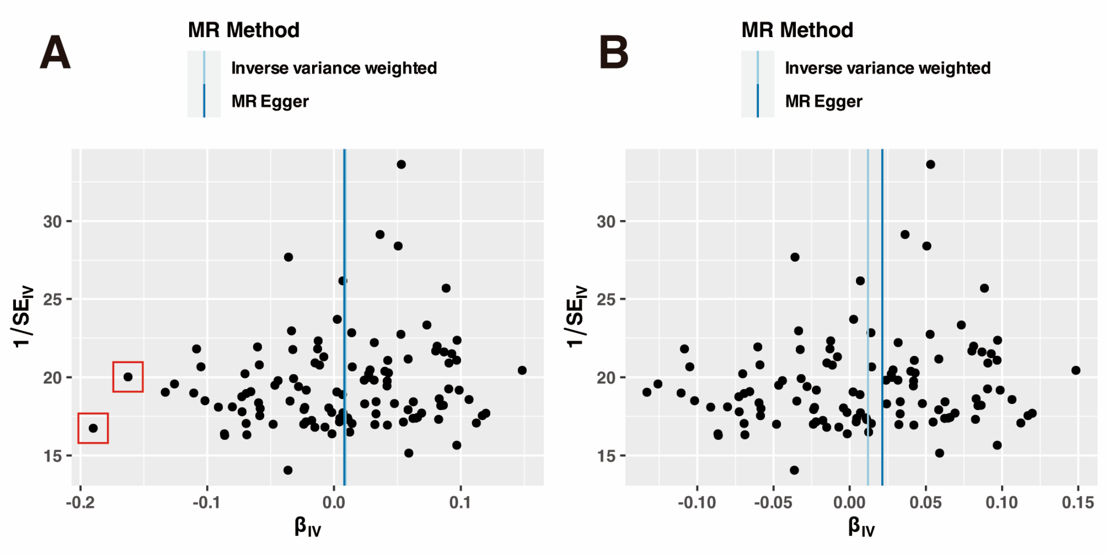

Supplementary Figure S6. Funnel plot for ADHD on calcium. (A) The funnel plot of ADHD-calcium results before removal of SNPs. (B) The funnel plot of ADHD-calcium results after removal of SNP rs34008721 and snp139950543.

The global MR pleiotropy residual sum and outlier (MR-PRESSO) test was introduced to explore the possible outlier SNPs. Each point is a single nucleotide polymorphism. ADHD, attention-deficit/hyperactivity disorder; SE, standard error.

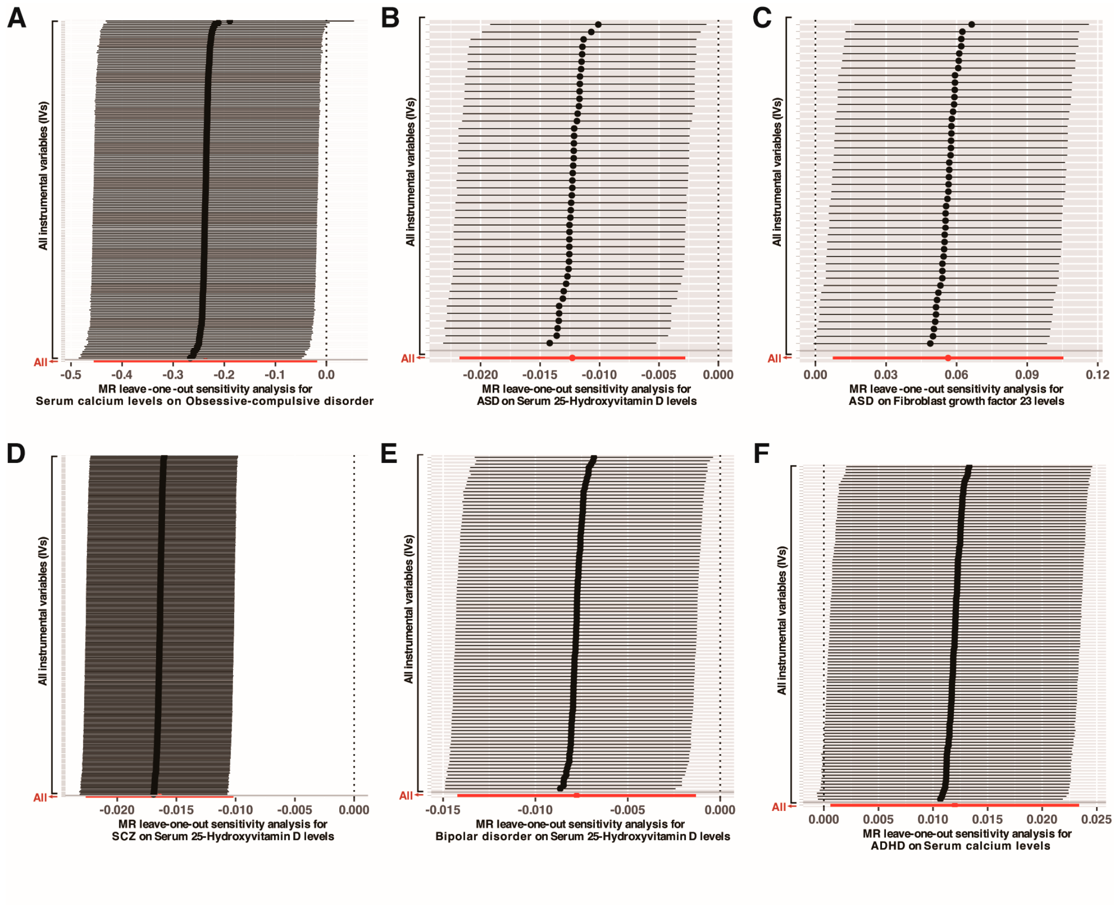

Supplementary Figure S7. Leave-one-out analysis of putative causal effects.

The x-axis shows the MR effect size of exposure on outcome. The y-axis shows the results of the analysis for each SNP. ASD, autism spectrum disorder; ADHD, attention-deficit/hyperactivity disorder; SCZ, schizophrenia.
